# Exosomes Regulate NLRP3 Inflammasome in Diseases

**DOI:** 10.3389/fcell.2021.802509

**Published:** 2022-01-03

**Authors:** Zhangwang Li, Xinyue Chen, Junjie Tao, Ao Shi, Jing Zhang, Peng Yu

**Affiliations:** ^1^ The Second Affiliated Hospital of Nanchang University, The Second Clinical Medical College of Nanchang University, Nanchang, China; ^2^ School of Medicine, University of Nicosia, Nicosia, Cyprus; ^3^ School of Medicine, St. George University of London, London, United Kingdom; ^4^ Department of Anesthesiology, The Second Affiliated Hospital of Nanchang University, Nanchang, China; ^5^ Department of Metabolism and Endocrinology, The Second Affiliated Hospital of Nanchang University, Nanchang, China

**Keywords:** exosomes, NLRP3, inflammasome, miRNA, diseases

## Abstract

Emerging evidence has suggested the unique and critical role of exosomes as signal molecules vector in various diseases. Numerous researchers have been trying to identify how these exosomes function in immune progression, as this could promote their use as biomarkers for the disease process and potential promising diagnostic tools. NOD-like receptor (NLR) family, pyrin domain containing 3 (NLRP3), a tripartite protein, contains three functional domains a central nucleotide-binding and oligomerization domain (NACHT), an N-terminal pyrin domain (PYD), and a leucine-rich repeat domain (LRR). Of note, existing studies have identified exosome as a novel mediator of the NLRP3 inflammasome, which is critical in diseases progression. However, the actual mechanisms and clinical treatment related to exosomes and NLRP3 are still not fully understood. Herein, we presented an up-to-date review of exosomes and NLRP3 in diseases, outlining what is known about the role of exosomes in the activation of NLRP3 inflammasome and also highlighting areas of this topic that warrant further study.

## Introduction

Exosomes exert a vital role in disease development (Kalluri and LeBleu, 2020). Exosomes (30–150 nm in diameter) arising from the membranes of multivesicular bodies (MVB) can transmit multiple biological molecules, thus regulating intercellular communication under normal and pathophysiological conditions (Pegtel and Gould, 2019). According to Vesiclepedia, a total of 349,988 proteins, 639 lipids, 27,646 messenger RNAs (mRNAs), and 10,520 microRNAs (miRNAs) have been found in exosomes. It has been revealed that exosomes could be used as a kind of disease biomarker to provide insights into the early diagnosis for cancers, such as gastric cancer, breast cancer, and prostate cancer (Jia et al., 2017; Fujita and Nonomura, 2018; Wang et al., 2018). Interestingly, the molecules in exosomes could reflect the features of the cells they come from to some extent. Compared with free biomolecules, the intrinsic features of biomolecules encapsulated by exosomes are more stable and biocompatible (Deb et al., 2021). Since exosomes were first discovered in 1983, their molecular mechanisms and functions have been increasingly explored (Zhang and Yu, 2019). It has been reported that exosomes greatly impact cancer progression, cardiovascular disease, liver fibrosis, non-alcoholic steatohepatitis (NASH), and metabolic diseases (Schroder et al., 2010; Wan et al., 20162016; Próchnicki and Latz, 2017; Moossavi et al., 2018; Tai et al., 2018; Zhou et al., 2018; Tao et al., 2020). Currently, there is a growing interest in the association between exosomes and immunoregulation mechanisms in diseases progression has been creeping up (Sharma and Johnson, 2020). For example, exosomes secreted by tumor cells have been highlighted in modulating the immune response in some cancer types (Greening et al., 2015). Emerging studies have confirmed that exosomes can enhance opsonization, modulate antigen presentation, and induce immune activation and immune suppression (Kalluri, 2016; Zhang and Yu, 2019).

NLRP3, a tripartite protein of the NLRP3 family of PRRs, oligomerizes to form the NLRP3 inflammasome upon activation serving as a pattern recognition receptor in the cytosol (Mangan et al., 2018; Swanson et al., 2019). NLRP3 inflammasome is widely present in immune cells. Its downstream effector proteins include caspase-1, interleukin-1β (IL-1β), and interleukin-18 (IL-18), which exhibit a protective or detrimental role in mucosal immunity (Zhen and Zhang, 2019). Of note, emerging evidence demonstrates that thioredoxin-interacting protein (TXNIP) is of great significance to NLRP3 inflammasome activation by the TXNIP-NLRP3 interaction (Zhou et al., 2010; Lerner et al., 2012; Mohamed et al., 2014). The association between TXNIP and NLRP3 was mainly linked to reactive oxygen species (ROS) accumulation. For example, in the progression of insulin resistance-related inflammation (Strowig et al., 2012), glucose could induce TXNIP expression, and ROS triggers its dissociation from thioredoxin, thus providing a signal for NLRP3 inflammasome activation and IL-1β release (Willeit et al., 2018).

Emerging studies have revealed the mechanisms through which exosomes modulate NLRP3 in diverse immunity processes (Bai et al., 2019; Tavakoli Dargani and Singla, 2019; Liang et al., 2020). Though the molecular functions in diseases have been extensively researched and the field has rapidly advanced, the complete regulation of exosomes and NLRP3, as well as clinical application, are still not fully understood. This review focused on the modulation mechanisms of the NLRP3 inflammasome by exosomes and the potential treatment targets in human diseases, hoping to bring and stimulate novel insights in clinical treatment.

## Characteristics and Functions of Exosomes in Inflammation

### Biogenesis and Function

Based on size, biogenesis, and content, extracellular vesicles (EVs) secreted by cells can be grouped into three main subtypes: apoptotic bodies, microvesicles, and exosomes (Doyle and Wang, 2019). Various investigations have revealed that both exosomes and microvesicles encapsulate specific sets of proteins, lipids, and nucleic acids, which can have a marked effect on intercellular communication as molecular agents (Simons and Raposo, 2009; Ludwig and Giebel, 2012; Raposo and Stoorvogel, 2013). However, there are no effective and precise separation methods to make exosomes distinguishable from other EVs (Ludwig et al., 2019). Apoptotic bodies (800–5,000 nm in diameter) and microvesicles (200–1,000 nm in diameter) are directly generated through the outward budding of the plasma membrane, whereas exosomes (30–150 nm in diameter) are vesicles of endocytic origin (van Niel et al., 2018). It has been reported that some vesicles (>150 nm in diameter) can also be secreted through an endosomal pathway and that other vesicles (<150 nm in diameter) can be directly formed from the plasma membrane (Nabhan et al., 2012). Therefore, the identification of exosomes based on size must be undertaken warily.

Many studies have confirmed the role of exosomes as carriers of biomarkers for diseases. The most commonly occurring membrane proteins include tetraspanins [e.g., cluster of differentiation 63 (CD63), cluster of differentiation 9 (CD9), and cluster of differentiation 81 (CD81)], heat shock proteins (e.g., Hspa8, Hsp90), GTPases [e.g., recombinant eukaryotic translation elongation factor 1 α 1 (EEF1A1), recombinant eukaryotic translation elongation factor 2 (EEF2)], and endosomal proteins and markers (e.g., Alix) (Ibrahim and Marbán, 2016; Gemel et al., 2019). Many efforts have been made to explore the specific and functional potential of exosomes. Yang et al. measured the level of the miRNA in Alzheimer’s disease and found that the serum exosome-derived miR-135a, miR-193b, and miR-384 could provide novel insights for the screening and prevention of Alzheimer’s disease (Yang et al., 2018a). It has also been reported that the miRNAs content of exosomes from the sputum is dysregulated in idiopathic pulmonary fibrosis (IPF), thus may be used as novel biomarkers for diagnosis (Njock et al., 2019). Moreover, exosomal signal regulatory protein α (SIRPα) proteins can effectively lead to phagocytic elimination of tumor cells (Koh et al., 2017). In prostate cancer (PC), fatty acid-binding protein 5 (FABP5) resulted as the potential significant exosomal-encapsulated protein in PC patients when compared to controls (Erozenci et al., 2019). In cancer immunotherapy, exosomes have gained lots of attention because of their functional roles in regulating immune responses (Xu et al., 2020).

For its transportation, immune cell-derived exosomes could contain various cargoes, including lipids and nucleic acids that are involved in both immune processes (Chaput et al., 2005). Their release into the extracellular milieu involves the fusion of the MVB with the plasma membrane. In addition, some of the proteins carried by exosomes include the major histocompatibility complex (MHC) and costimulatory vesicles, which ultimately participate in exosome-induced regulation of immune responses. The cargoes of exosomes originate from the Golgi apparatus or the plasma membrane and are sorted into MVBs before being released as intraluminal vesicles (ILVs) (van Niel et al., 2018).

### Inflammation Regulation

Over recent years, exosomes have been considered pivotal signal molecules vector in inflammatory processes that transfer proteins, lipids, and nucleic acids, thus influencing the target cell’s metabolism in many diseases, including cancer, cardiovascular disease, and neurodegenerative disorder (Zhang et al., 2019a; Console et al., 2019; Zhou et al., 2020a; Bouchareychas et al., 2020). For example, exosome-mediated delivery of miR-155, miR-124-3p, miR-138 are involved in acute lung inflammation, traumatic brain injury, and endometriosis, respectively (Huang et al., 2018; Zhang et al., 2019b; Jiang et al., 2019). It has been found that the liposome‐like nanoparticles (LLNs) assembled with lipids from Ginger ELNs (GELNs) are of significance in intestinal stem cells (Ju et al., 2013). Meanwhile, it has been reported that lipid carried by exosomes may help to reinstate lipid raft functions and restore barrier integrity in inflammatory bowel disease (Bowie et al., 2012). Experiments associated with exosomal-encapsulated proteins in inflammation have also been conducted. Macrophage exosomes can deliver protein to the inflamed brain, thus affecting inflammation (Yuan et al., 2017). S100 Calcium Binding Protein A9 (S100-A9) protein in exosomes can promote inflammation in polycystic ovary syndrome (Li et al., 2020). It has been noted that interleukin-35 encapsulated by exosomes may be a critical molecular for suppressing inflammatory responses (Kang et al., 2020).

Recently, there have been more and more studies on the regulation of NLRP3 by exosomes inflammatory mechanisms (Bai et al., 2019; Liang et al., 2020; Si et al., 2021). For example, Yang et al. found that umbilical cord mesenchymal stem cells-derived exosomes (UMSC-Exo) attenuated production of cleaved caspase-1 and subsequently decreased IL-1β and IL-18 release and pyroptosis. circHIPK3 released by UMSC-Exo down-regulated miR-421, resulting in increased expression of fork head box class O 3a (FOXO3a), which could inhibit NLRP3 activation (Yan et al., 2020). Herein, we discussed the relationship between the exosomes and NLRP3.

## Characteristics and Pathological Functions of NLRP3 Inflammasome

NLR protein family has 22 members in humans and at least 34 members in rodents (Ting et al., 2008). As a unique receptor, NLRP3 is the best characterized and the most extensively studied among the NLRP3 family (Yang et al., 2019). NLRP3 consists of three functional domains: an amino-terminal PYD, a NACHT, and a LRR (Swanson et al., 2019). Accumulating evidence has reported that NLRP3 inflammasome has a critical role in inflammatory diseases (Mangan et al., 2018).

NLRP3 inflammasome is a high-molecular-mass protein complex consisting of an upstream sensor protein NLRP3, a downstream effector protein caspase-1, and an apoptosis-associated speck-like protein containing a CARD (ASC) (Lamkanfi and Dixit, 2012). Of note, NIMA related kinase7 (NEK7), a newly demonstrated NLRP3-binding protein, regulates NLRP3 oligomerization and activation by forming a supramolecular NLRP3-NEK7 complex (He et al., 2016a). ASC has two domains, containing PYD and C-terminal caspase recruitment domain (CARD), which can interact with other proteins (Lu et al., 2016). ASC combines with NLRP3 by homotypic PYD-PYD interactions, after which it recruits pro-caspase-1 through CARD-CARD interactions forming the NLRP3 inflammasome (Cai et al., 2014; Sharif et al., 2019). Then, the dormant pro-caspase-1 is cleaved into active caspase-1, which trims pro-IL-1β and pro-IL-18 to generate mature and active cytokines IL-1β and IL-18 (Mangan et al., 2018). Caspase-1 also cleaves pro-gasdermin-D (GSDMD) to generate an N-terminal fragment, after which it forms GSDMD pores, controlling the release of IL-1β and IL-18 (Rathinam et al., 2019). This enables cytokine secretion, water influx, osmotic swelling, and cell ruptures, eventually leading to pyroptosis (Liu et al., 2016; Orning et al., 2019; Liu et al., 2020).

To the best of our knowledge, typically in most cell types, the activation of NLRP3 occurs requiring a minimum of two steps: Priming and Activation (Xue et al., 2019a; Wang and Hauenstein, 2020). Priming is the first step initiated by various damage associated molecular patterns (DAMPs) and pathogen-associated molecular patterns (PAMPs) that are recognized by Toll-like receptors (TLRs), activating the nuclear factor κB (NF-κB) signaling pathways (Gritsenko et al., 2020). NF-κB could enhance the transcription of pro-IL-1β, pro-IL-18, and NLRP3 (Afonina et al., 2017). Furthermore, the second step (activation) is the oligomerization of NLRP3 and the assembly of NLRP3, NEK7, ASC, and pro-caspase-1 into the NLRP3 inflammasome (Strowig et al., 2012), which is mostly triggered by adenosine triphosphate (ATP) (Karmakar et al., 2016), purinergic ligand-gated ion channel 7 receptor (P2X7R) (Wang et al., 2020), mitochondrial reactive oxygen species (mtROS) (Zhong et al., 2016), mitochondrial damage (Zhong et al., 2016), autophagic dysfunction (Xue et al., 2019b), dynamin-related protein 1 (Drp1) (Wang et al., 2014), oxidized mitochondrial DNA (ox-mtDNA) (Jia et al., 2020a), mitochondrial abtiviral-signaling protein (MAVS) (Subramanian et al., 2013) and so on (Tschopp and Schroder, 2010; Seoane et al., 2020).

**FIGURE 1:**
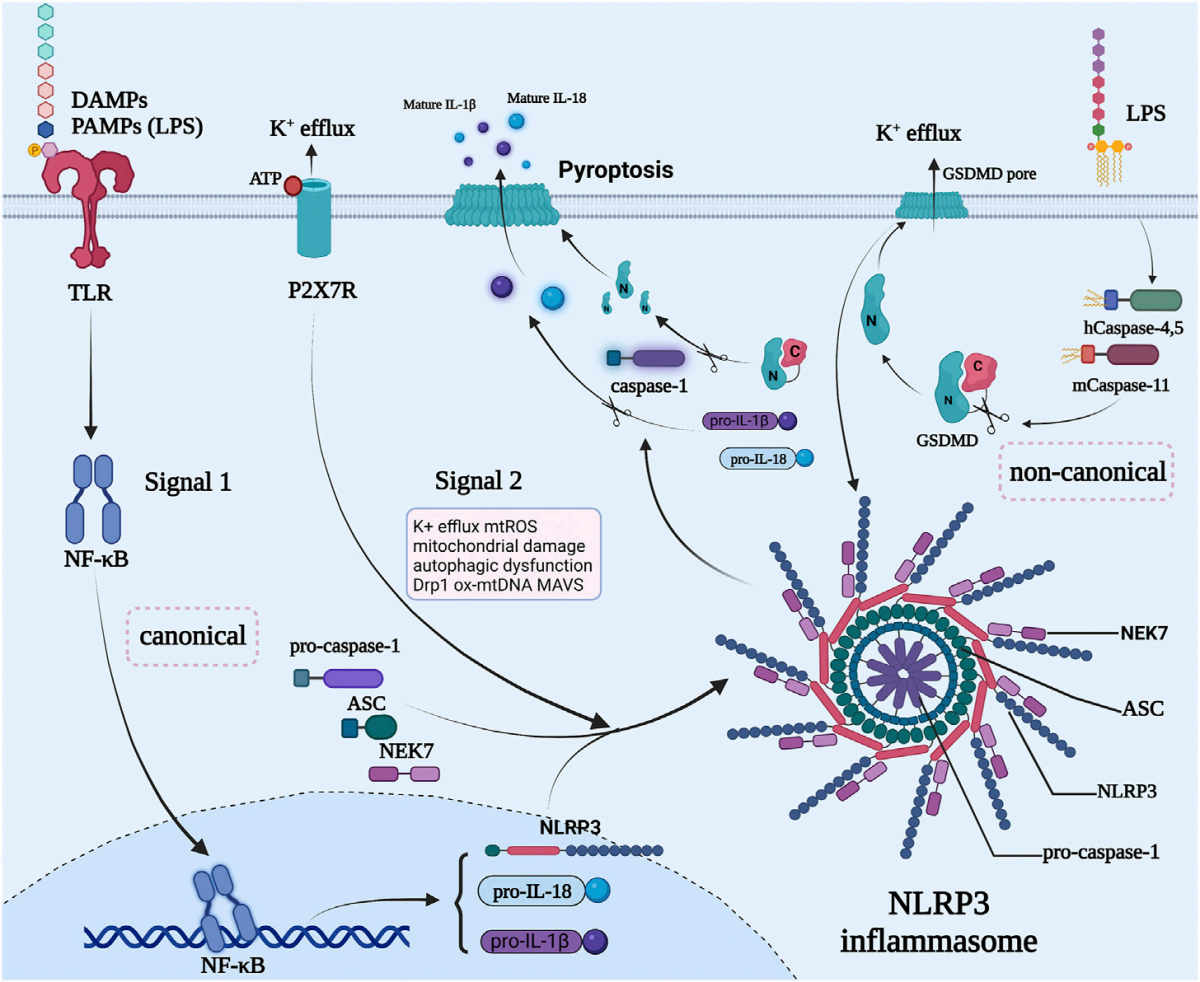
Canonical and non-canonical NLRP3 inflammasome pathways. NLRP3 inflammasome is activated through a process, which involves a minimum of two steps in most cell types. First, a priming step is required by treating cells with a toll-like receptor ligand like lipopolysaccharides (LPS), which activates the NF-κB pathway to induce the transcription of NLRP3, caspase-1, and pro-IL-1β. Signal 2 is provided by PAMPs or DAMPs that activate multiple events mainly induced by potassium efflux. The inflammasome is formed by assembling NLPR3, NEK7, ASC, caspase-1. Next, the activated NLRP3 inflammasome acts as a platform for the processing of caspase-1, whose main function is the conversion of the inactive cytokines pro-IL-1β, pro-IL-18, and GSDMD into their active forms. Non-canonical NLRP3 inflammasome activation is triggered by the cytosolic LPS sensing by caspase 4/5/11 and then they cut their substrates GSDMD, resulting in the formation of GSDMD membrane pores, causing potassium efflux, which promotes the activation of NLRP3 inflammasomes. The activated NLRP3 inflammasome further cuts the GSDMD to form more membrane pores and induce the maturation of pro-IL-1β, pro-IL-18, and caspase-1, causing pyroptotic cell death. The figure was constructed with BioRender (https://biorender.com/).

Currently, numerous studies have revealed that NLRP3 inflammasome has a crucial role in the pathogenesis of many diseases (Koka et al., 2017; Huang et al., 2021), such as cancer (Bruchard et al., 2013), cardiovascular diseases (Liu et al., 2018a), and metabolic diseases (Sharma and Kanneganti, 2021). NLRP3 inflammasome activation is induced by three different pathways in different cells, including canonical pathway, non-canonical pathway, and alternative pathway (Figure 1).

## Exosome and NLRP3 Inflammasome Activation Pathways

**FIGURE 2:**
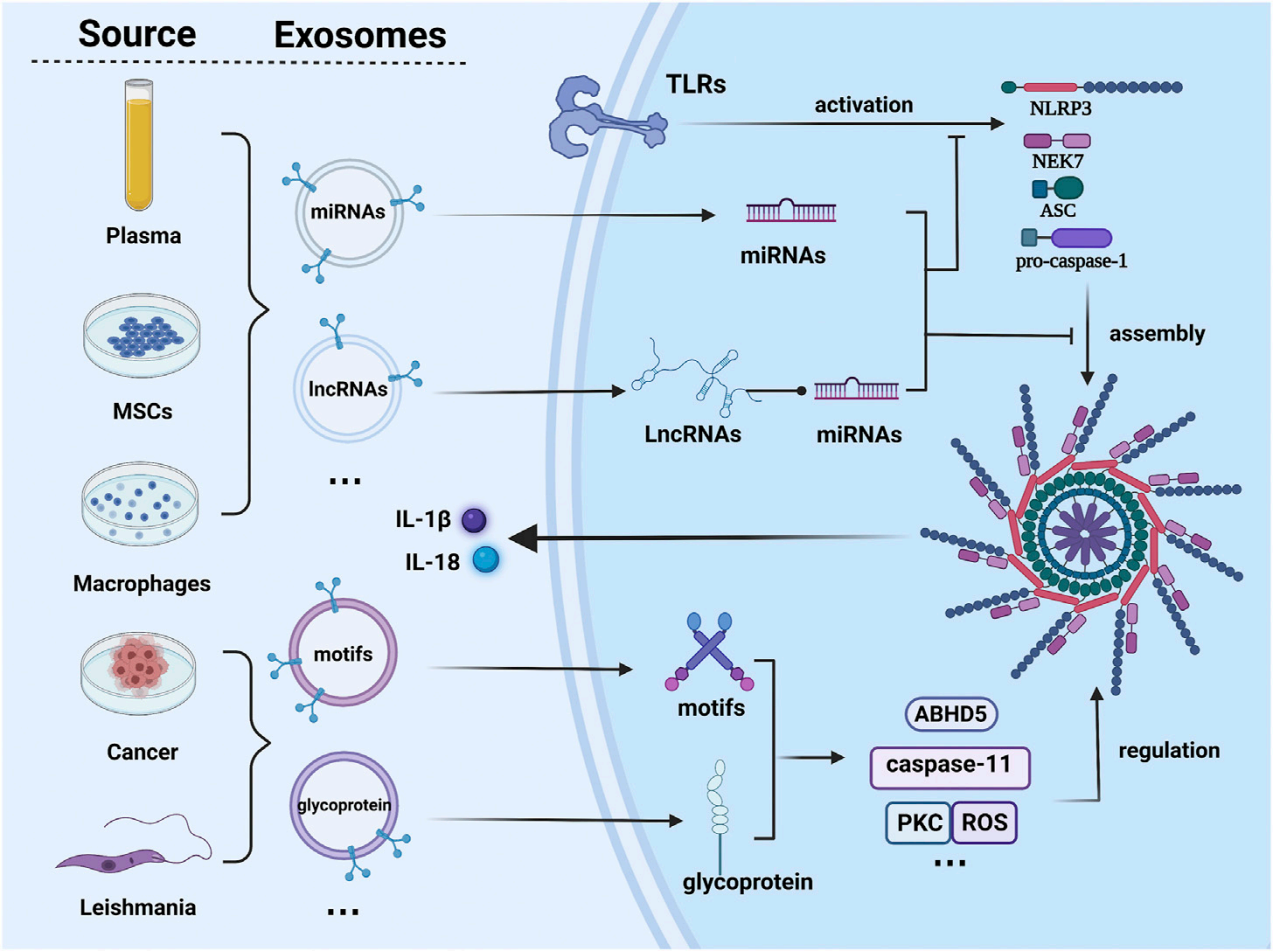
Different sources-derived exosomes regulate the NLRP3 pathway in various patterns. Exosomes from plasma, macrophages, and mesenchymal stem cells suppress the activation of the NLRP3 pathway or the expression of the key molecule from the related signaling axis to regulate the progression of diseases. Otherwise, cancer cells and some pathogens may behave in another way. They release particular exosomal antigens and morbid substances to model a favorable environment for their own survival. Several pathogenic patterns were found. lncRNA, long non-coding RNA; miRNA, microRNA; PKC, Protein Kinase C. The figure was constructed with BioRender (https://biorender.com/).

The crosstalk between exosome and NLRP3 inflammasome has been demonstrated with converging evidence in recent years. The inhibition of exosomal programmed death-ligand 1 (PD-L1) can result in systemic anti-tumor immunity (Poggio et al., 2019). Exosome-signal regulatory protein α (SIRPα) can interact with cluster of differentiation 47 (CD47) exiting in the tumor cells surface, thus restricting macrophages from engulfing tumor cells (Koh et al., 2017). Several experiments have revealed that exosomes can modulate immunity in diseases by monitoring programmed cell death-1 (PD1), gp130/STAT3 signaling, and the TLR4/NF-κB/NLRP3 inflammasome (Yang et al., 2018b; Ham et al., 2018; Dai et al., 2020). An increasing body of evidence reveals that exosomes are involved in the occurrence and development of diseases through NLRP3 inflammasome canonical and non-canonical pathways (Shi et al., 2020; Cai et al., 2021). Remarkably, emerging studies have uncovered the potential regulatory role of NLRP3 inflammasome activation on exosome production. Based on the available evidence, exosomes were demonstrated to exert dual effects on inflammasome activation (Noonin and Thongboonkerd, 2021). Promotion of inflammation (Liang et al., 2020) or control of pathogenesis (Singla et al., 2019) can have both positive and negative aspects, which depend on the molecular composition of exosomes (Figure 2). Therefore, a better understanding of the relationship between exosome and NLRP3 inflammasome activation may reveal a potential target for the therapy of NLRP3 inflammasome-related diseases.

### Exosomes and the Canonical NLRP3 Activation Pathway

Previous studies have reported that NLRP3 inflammasome is activated by a minimum of two steps in most cell types (Sutterwala et al., 2006; Bauernfeind et al., 2009). First, a priming step is required by treating cells with TLR ligands, DAMPs and PAMPs like LPS, which activates the NF-κB pathway to induce the transcription of NLRP3, caspase-1β, and pro-IL-18 (Sutterwala et al., 2006; Lu et al., 2014). This is followed by treating cells with “danger signal” molecules, such as mtROS, mitochondrial damage, autophagic dysfunction, Drp1, ox-mtDNA, MAVS, and so on (Elliott and Sutterwala, 2015; He et al., 2016b; Jo et al., 2016; Zhou et al., 2020b; Weber et al., 2020), which leads to multiple signaling events mainly induced by potassium efflux. These events trigger the assembly of NLRP3 inflammasome (Cai et al., 2014) that manifests as a large perinuclear ASC speck in the cell (Masumoto et al., 1999). Next, the activated NLRP3 inflammasome acts as a platform for the processing of caspase-1. After processing, the pro-caspase-1 becomes the functional caspase-1, whose main function is the conversion of the inactive and intracellularly potent pro-inflammatory cytokines pro-IL-1β, pro-IL-18, and the pore-forming protein GSDMD) into their active forms (Miao et al., 2006; Jo et al., 2016). Mature IL-1β and IL-18 are then secreted from the cells (Dinarello, 2009), leading to pyroptosis. Emerging evidence has shown that exosomes can affect the key molecules in the canonical pathway by secreting different substances, which in turn affects the process of NLRP3 inflammasome-related disease (Yan et al., 2020; Li et al., 2021; Si et al., 2021; Wang et al., 2021). Dai et al. found that miR-148a enhanced cell viability and relieved cardiac enzymes dysregulation and Ca2+ overload in ischemia/reperfusion-induced neonatal rat cardiomyocytes (NCMs). M2-exos-carried miR-148a could directly target TXNIP and obviously inhibited the expression of TXNIP, significantly inhibiting TLR4 expression. The downstream NF-κB activation and NLRP3 inflammasome components expression, therefore, were markedly suppressed, which alleviated myocardial ischemia/reperfusion injury (Dai et al., 2020). Meanwhile, Yan et al. reported that circular homeodomain-interacting protein kinase 3 (circHIPK3) from UMSC-Exo can downregulate miR-421, resulting in the upper expression of FOXO3a, thus preventing the activation of NLRP3 inflammasome and caspase-1 prevent pyroptosis and repairing ischemic muscle injury (Yan et al., 2020).

### Exosomes and the Non-Canonical NLRP3 Activation Pathway

Unlike the canonical pathway, NLRP3 inflammasomes can also be activated by Human caspase-4,5 or Murine caspase-11 directly sensing the cytosolic LPS from Gram-negative bacteria (Shi et al., 2014), then the caspase-4/5/11 could then cut their substrate GSDMD, resulting in the formation of GSDMD membrane pores (Ding et al., 2016). Furthermore, potassium efflux caused by GSDMD membrane pores promotes the activation of NLRP3 inflammasomes. The activated NLRP3 inflammasomes further cut the GSDMD to form more membrane pores and induce the maturation and secretion of IL-1β, IL-18, and caspase-1, eventually causing pyroptotic cell death (Bossaller et al., 2012; Antonopoulos et al., 2015; Komada and Muruve, 2019). According to the literature, there are no reports of studies noted that exosomes could regulate the non-canonical NLRP3 inflammasome activation pathway to influence the development of diseases. However, the non-canonical NLRP3 inflammasome activation pathways exert significant clinical ramifications (Downs et al., 2020), thus more studies are urgently needed to uncover the mechanisms underlying the regulation of exosomes on the non-canonical NLRP3 inflammasome activation to explore potential therapeutic targets.

Figure 1 Different sources-derived exosomes regulate the NLRP3 pathway in various patterns. Exosomes from plasma, macrophages, and mesenchymal stem cells suppress the activation of the NLRP3 pathway or the expression of the key molecule from the related signaling axis to regulate the progression of diseases. Otherwise, cancer cells and some pathogens may behave in another way. They release particular exosomal antigens and morbid substances to model a favorable environment for their own survival. Serval pathogenic patterns were found. PKC, Protein Kinase C; miRNA, microRNA; lncRNA, long non-coding RNA.

### NLRP3 Downstream Exosomes Release—Inflammation and More Than Inflammation

The activation of the NLRP3 inflammasome could result in the maturation and exosomal release of IL-1β, IL-16, and ASC (Qu et al., 2009; Dubyak, 2012), thus exosomes exert a critical role in forming the pro-inflammatory status in tissues. However, the NLRP3-promoted exosomes secretion seems not limited to loading inflammatory cytokines. A recent review of exosomes in helping α-synuclein transmission in Parkinson’s disease (PD) suggests that NLRP3 could influence diseases not only by inducing inflammatory status and pyroptosis but in a way of facilitating the exosomal release of specific pathological proteins (Si et al., 2021). The elevated membrane-shed exosomes could even load protective molecules and chemo-active compounds, like nitric oxide aganist cardiovascular damage (Pearce et al., 2021). Therefore, exosomes exert as “a double-edged sword” in inflammation.

NLRP3 regulating exosomes in diseases can be interestingly affected by a wide range of chemokines. Sarkar et al. found that Mn^2+^ can induce cell-to-cell transfer of the inflammasome adaptor protein ASC in exosomes, which might be a potential mechanism in neurodegenerative diseases (Sarkar et al., 2019). Carbon monoxide can activate the NLRP3 pathway and the secretion of exosomes (Chen et al., 2021). Betaine inhibits the extracellular secretion of secretory lysosomes containing IL-1β, then inhibits the extracellular secretion of IL-1β-containing exosomes, thereby inhibiting IL -1β release (Xia et al., 2018).

## The Regulatory Mechanisms of Exosomes to NLRP3 Inflammasome in Diseases

NLRP3 inflammasomes act as a platform for activating cytokines like IL-1β, and is a key meditator of the inflammatory response (Zamboni and Lima-Junior, 2015). In host-response and pathogens resistance, the inflammasome-induced response has been proven to be of great importance. Moreover, the modern lifestyle has brought new challenges to healthcare, including the problem of chronic diseases hallmarks. The reprogramming of innate immune cells and persistent activation of inflammasomes have been identified as essential in the mechanisms underlying an array of non-communicable diseases, thus making inflammasome a promising target for therapeutic use.

The model of NLRP3 inflammasome activation varies among different types of diseases. Potassium efflux and NEK7, which directly interact with NLRP3 and assist its activation, seem to be the conserved elements in NLRP3 activation (He et al., 2016a; Shi et al., 2016). Exosomes participate in the regulation of the whole process of NLRP3 activation. It has been reported that exosomes can carry the antigens or non-coding RNAs, which in turn could influence the expression of some key molecules (Liu et al., 2018b; Cai et al., 2021). For example, stem cells-derived exosomes can reverse the immune cell reprogramming and inflammatory state by correcting the over-activation of NLRP3. In addition, exosomes which targeting NLRP3 and its downstream IL-1β offer an anti-inflammation therapy method without increasing the risk of infection, as anti-IL-1β therapy might directly induce immunosuppression (Ridker et al., 2017). The summary of current exosomal NLRP3 regulation in diseases is presented in Table 1.

**TABLE 1:** Source of exosomes and roles in regulation of NLRP3-related diseases.

Disease	Exosomes source	Pivotal molecules	Role of the exosomes	Ref.
Cancer	Lung cancer cells	TRIM59	TRIM59/ABHD5/NLRP3 signaling axis	Lerner et al. (2012)
	M2 macrophages	miR-223	Repressing the canonical NF-κB and TLR-9- NLRP3 pathway	Becatti et al. (2012), Mao et al. (2019)
	T24 cells, SV-HUC-1 cells	miR-375-3p	A bladder cancer suppressor via Wnt/β-catenin pathway	Vandanmagsar et al. (2011)
Ischemia/Reperfusion injury	Mesenchymal stem cells	miR-320b	Inhibitor of the expression of NLRP3 gene	Elliott and Sutterwala (2015)
	Plasma	miR-148a	Reduction of myocardium damage via TXNIP/NLRP3/caspase-1 pathway	Saberi et al. (2009)
	Mesenchymal stem cells	LncRNA KLF3-AS1	Sponge of miR-138-5p, which can overexpress SIRT, then inhibit NLRP3	Weber et al. (2020)
	Umbilical cord stem cells	miR-26b-5p	Suppressing the polarization of M1 macrophages leading to the downregulation of TLR-2,4,6	Zhang et al. (2021a)
	Adipose stem cells	Unidentified miRNAs	Inhibition of Wnt/β-catenin signaling pathway	Zhang et al. (2019c)
Myocardial infarction	Umbilical cord stem cells	miR-100-5p	Downregulation of FOXO3 translation to block NLRP3 activation	Incalza et al. (2018)
Carotid endarterium injury	Adipose stem cells	Pre-STC1	Low-expression of NLRP3 inflammasome	Pajukanta et al. (2004)
Dox cardiomyopathy	Embryonic stem cells	Anti-inflammation cytokines	Inducing M2 macrophages polarization and releasing of IL-10	Gritsenko et al. (2020)
Diabetes	Mesenchymal stem cells	miR-126	Downregulation of HMGB1, which can stimulate TLR4-NLRP3	Zhang et al. (2021b)
	Adipose stem cells	Unidentified miRNAs	Inhibitor of ROS-TXNIP-NLRP3 pathway	Wang et al. (2021)
	Pericytes, endotheliocytes	circEhmt1	Regulation of high glucose microvascular dysfunction via the NFIA/NLRP3 pathway	Bechard et al. (2020)
Atherosclerosis	Plasma	miR-223	Inhibitor of the expression of NLRP3 gene	Xue et al. (2019a)
Pulmonary fibrosis	Endometrial stem cells	miRNA Let-7	Regulating mtDNA damage, repressing LOX1/NLRP3/caspase 3	Zhang et al. (2021c)
Leishmaniasis	Leishmania	GP63	Influencing HZ-induced NLRP3 pathway activation and cleaving the inflammasome complex	Ridker et al. (2017)
	Leishmania	LPG	Involving in activation of non-canonical pathway of NLRP3	Minutoli et al. (2016)
HIV-1	Human bone marrow derived macrophages	HIV protein Nef	Redistributing TLR4 toward lipid rafts	Tang et al. (2020)
Gastrointestinal helminth	Worms	micro-RNAs	Enhancing the NLRP3-dependent IL-18 and IL-1β secretion	Kore et al. (2021)
Sepsis	M1 and M2 macrophages	miR-93-5p	Targeting on TXNIP and influence the activation of NLRP3	Haneklaus et al. (2013)
Muscle ischemia	Umbilical cord stem cells	miR-29b	Binding to cPWWP2A and regulate the PWWP2A/Rb1/AMPKα2/NLRP3 signaling pathway	Bruchard et al. (2013)
Parkinson’s disease	Adipose stem cells	miR-188-3p	Suppression of NLRP3 and CDK5	Zhang et al. (2020)

TRIM59, tripartite motif-containing 59; ABHD5, abhydrolase domain containing 5; NLRP3, NLR family protein containing a pyrin domain 3; NF-κB, nuclear factor κB; TLR, toll-like receptor; SV-HUC-1 cells, human bladder cell biochemistry pillon; Wnt/β-catenin, wingless/β-catenin; LncRNA, long non-coding RNA; SIRT, sirtuin; FOXO3, forkhead box O3; STC-1, stanniocalcin-1; IL, interleukin; HMGB1, high-mobility group protein 1; ROS, reactive oxygen species; TXNIP, thioredoxin-interacting protein; NFIA, a transcription factor; mtDNA, mitochondrial RNA; LOX1, lectin-type oxidized LDL receptor 1; AMPKα2, AMP kinaseα2; CDK5, cyclin dependent kinase 5.

### Exosomal Anti-Pyroptosis via NLRP3 Inflammasome in Cardiovascular Diseases

Pyroptosis is a newly founded cell death process characterized by excessive inflammatory cytokines release, which closely associated with the NLRP3-GSDMD pathway. In ischemia/reperfusion (I/R) injury models, NLRP3, caspase-1, and GSDMD are notably heightened (Jia et al., 2020b). It has been reported that mitochondrial dysfunction and ROS accumulation are crucial in I/R-related NLRP3 activation (Minutoli et al., 2016; Gong et al., 2018; Jia et al., 2020b).

Of note, human bone marrow mesenchymal stem cell (MSC)-derived dramatically suppresses the expression of NLRP3 in the I/R myocardium. Tang et al. discovered that MSC exosomal miR-320b could directly target the NLRP3 molecule, then negatively regulate the downstream expression of the caspase-1, thus inhibiting the pyroptosis in the rat myocardial I/R model (Tang et al., 2020). Results of recent proteomics research between infract and pre-infract myocardium provided support for the protective function of human MSC-derived exosomes against cell pyroptosis (Kore et al., 2021). Damage to the ischemic myocardium can be reduced by exosomal miR-148a via inhibiting the TXNIP-NLRP3-caspase-1 pathway (Becatti et al., 2012). Sirtuin 1 (SIRT1) is a potent protector from aging-associated-pathologies. Furthermore, SIRT1 was identified at a critical point in exosomal NLRP3 regulation. Long non-coding RNA (lncRNA) KLF3-AS1, a molecular sponge of miR-138-5p, was discovered at a vital point in exosomal NLRP3 regulation (Mao et al., 2019). Highly concentrated miR-138-5p can upregulate the expression of SIRT1, which has a critical role in NLRP3 auto-inhibition, thus decreasing the myocardial infarction-induced damage (Mao et al., 2019).

Over recent years, many studies reported that doxorubicin (DOX) cardiotoxicity could be alleviated through embryonic stem cell-derived exosomes (ES-Exo) therapy. Pyroptosis and inflammation have been found to be linked to DOX-induced cardiac remodeling and cell death, which can be alleviated by ES-Exo (Tavakoli Dargani and Singla, 2019). Singla and others first demonstrated that ES-Exo-induced M2 macrophages polarization and subsequent TLR4-NLRP3 downregulation were involved in exosomal ameliorating DOX-induced pyroptosis (Singla et al., 2019). Yet, more specific data on the underlying mechanism are warranted.

### Exosomes-Meditated NLRP3 Sensing in Metabolic Diseases

Previous studies have suggested that NLRP3 inflammasome does not merely work as a pro-inflammatory mediator but also as an unexpected sensor for metabolic stress. Cholesterol accumulation can give the priming signal for NLRP3 activation (Vandanmagsar et al., 2011). By directly interacting with TLR4, subsequently formed cholesterol crystal can also stimulate the NLRP3 inflammasome activation afterward (Saberi et al., 2009). The persistent activation of NLRP3 results in chronic inflammation, one of the key mechanisms in various metabolic diseases, which could be alleviated by ES-Exo. As a vector of intercellular genetic exchange, ES-Exo can downregulate the chronic NLRP3 activation at the cellular expression level, thus acting as a promising therapy carrier toward long-term inflammatory states.

Hyperglycemia-induced retinal inflammation and diabetic osteoporosis can be alleviated by ES-Exo (Zhang et al., 2021a). Previous studies have indicated that MSC-Exo-derived miR-126 can suppress hyperglycemia-induced inflammation by down-regulating high mobility group box-1 (HMGB1) protein, which can bind to TLR2, TLR4, and inflammasomes then help activate the NLRP3 pathway (Zhang et al., 2019c). HMGB1 is actively generated in human retinal endothelial cells and released after retinal damage or pathogenic changes. Diabetic limb ischemia is characterized by continuous ischemia and hyperglycemia in the muscle microenvironment. In addition, constant metabolic disorders and hyperglycemia in the tissue are expected to result in inflammation and oxidative stress in the tissue (Incalza et al., 2018).

TXNIP was at first in metabolism diseases identified as a key molecule of NLRP3 (Pajukanta et al., 2004). However, more recently published studies showed that TXNIP is extensively related to varieties of chronic inflammation in categories of different diseases, such as Alzheimer’s diseases and pancreatic cancer (Bechard et al., 2020; Zhang et al., 2021b), which suggests that TXNIP might be a promising vital meditator in the complex signal conduction network among oxidative stress, mitochondrial stress, ROS accumulation, and NLRP3 inflammasome-induced pyroptosis. Zhang et al. found that adipose-stem-cells-derived exosomes can improve endothelial cells function in diabetic limb ischemia. The inhibition of the ROS-TXNIP-NLRP3 pathway by miRNAs derived from exosomes was inferred as a major inner mechanism; however, further sequencing of miRNAs derived from the exosomes is needed (Zhang et al., 2021c).

Hyperlipidemia is a significant risk factor inducing atherosclerosis. Paeonol, as a promising drug for atherosclerosis, can raise the expression level of plasma-derived exosomal miRNA-223 in rats (Shi et al., 2020). miRNA-223 is an inhibitor of NLRP3 inflammasomes forming, whose upregulation can notably delay the progression of chronic inflammation in atherosclerosis (Haneklaus et al., 2013).

### Cancer-Derived Exosomes in Orchestrating the Polarization of TAMs Through NLRP3

Metabolic reprogramming of tumor-associated macrophages (TAMs) has a terminal role in cancer progression and resistance (Zhang et al., 2020). There is increasing evidence suggesting that exosomes, as a novel cellular communication mechanism, are engaged in malignant cells orchestrating tumor microenvironment (TME) via TAMs. cancer-derived exosomes can reportedly cause polarization in TAMs, thus increasing IL-1β secretion toward the microenvironment (Atay et al., 2011; Bardi et al., 2018; Li et al., 2018), which is related to poor clinical prognosis. The heightened exosome-induced IL-1β secretion facilitates the formation of a pro-inflammatory state in TME, which has been found among various solid neoplasms in previous studies (Oh et al., 2016; Wu et al., 2016; Dmitrieva-Posocco et al., 2019). Immune cells secreting IL-1β depend on the activation of NLRP3 inflammasomes. Interestingly, both cancer progression and lung metastasis can be exhibited by ultraviolet-irradiated apoptotic cancer cells through enhancement of the PPARγ/PTEN signaling in macrophages (Kim et al., 2019). Thus, the effects induced by cancer cells-derived exosomes are heterogeneous.

TAMs are highly plastic and can rapidly adapt to micro-environmental changes (Andrejeva and Rathmell, 2017). They sensitively detect the signals transported by exosomes and then genetically reprogram their function and phenotypes. NLRP3 pathway widely exists in innate immune cells and is highly expressed in immunosuppressive and inflammatory microenvironments. Meanwhile, cancer cells can directly or indirectly affect the key molecules in the NLRP3 pathway, thus leading TAMs reprogramming toward a pro-inflammatory state. Liang et al. reported murine lung cancer-derived exosomes, which express tripartite motif-containing 59 (TRIM59) and can regulate the NLRP3 pathway, intermediated by upregulating abhydrolase domain containing 5 (ABHD5) (Liang et al., 2020). TRIM59 directly induces the ubiquitination of ABHD5, leading to its proteasome-dependent degradation, activating the NLRP3 inflammasome signaling pathway, and promoting the secretion of IL-1β by macrophages; As the final result, exosomal TRIM59 facilitates lung cancer growth and metastasis. Besides, ABHD5 is an important regulator in lipid metabolism, whose deficiency has been shown to be a permission signal of the reprogramming of macrophages and the activation of the NF-κB-dependent NLRP3 inflammasomes pathway (Shang et al., 2019).

The polarization of TAMs exerts a role in cancer progression. M1-like TAMs have a tendency toward forming proangiogenic and pro-inflammatory states (Chen et al., 2011), and M2-like TAMs tend to engage in tissue remodeling. It has been reported that miRNA-223 can mediate the inflammatory response in macrophages by suppressing the canonical NF-κB pathway, which is related to the expression of NLRP3 (Liu et al., 2015). Endogenous miRNA-223 can be transported by exosomes toward hepato-carcinoma cells to inhibit tumor proliferation (Aucher et al., 2013). Based on this, it has been suggested that exosomes may serve as a potential therapeutic target among various cancers. In addition, with a current hypothesis, further studies are needed to validate the precise mechanism of exosome-mediated-NLRP3 inflammasome activities in cancer progression.

### Exosomes, NLRP3, and Host Defense

Leishmania parasites infect the macrophages and inhibit the production and release of several pro-inflammatory cytokines, e.g., IL-1β and IL-18 suppress the host defense and hence survive. The critical virulence factor of Leishmania--metalloprotease glycoprotein 63 (GP-63) can exhibit the PKC pathway, which is of vital importance for both ROS generation and the downstream NLRP3 signaling activation (Cosentino-Gomes et al., 2012; Shio et al., 2015). Exosomes contribute a lot as a major transporter of GP-63. GP-63 has been shown to promote the cleavage of a wide variety of cell substrates, of which cell phosphatase is the most evident. Interestingly, TXNIP, which has been shown to promote the activation of ROS-dependent inflammasomes, was identified as one of the GP-63-cleaved proteins (Shio et al., 2015).

Moreover, the endosymbiotic virus Leishmania RNA Virus 1 virus co-infection can potentiate the interference to the NLRP3 inflammasome network induced by exosomal GP-63 (Olivier and Zamboni, 2020). Membrane glycoconjugate lipophosphoglycan from Leishmania parasites stimulates macrophages through caspase-11, therefore, triggering the non-canonical NLRP3 activation, which is conducted in a pro-inflammatory way instead of an immunosuppressive way (de Carvalho et al., 2019). Limited evidence suggested that it might be transported to the host macrophage’s cytoplasm by exosomes before or after the Leishmania invasion (Silverman et al., 2010).

According to previous *in vivo* research, exosomal HIV-1 protein Nef can prone to induce the activation and redistribution of TLR4 in raft lipid (Mukhamedova et al., 2019). Also, the occurrence of subsequent NLRP3 signal cascade and downstream inflammatory response elevation has been confirmed (Mukhamedova et al., 2019). Worm exosomes were associated with NLRP3-mediated IL-18 secretion in gastrointestinal infection (Alhallaf et al., 2018).

### Therapeutic Potentials of Exosomes on NLRP3

Exosomes as carriers for drugs are being actively researched. Compared to liposomes, injected exosomes can effectively invade other cells and perform functions with minimal immune elimination (Kalluri, 2016; Fitts et al., 2019). Moreover, exosomes are more promising for therapeutic use as they have proven to be well tolerated. Repeated injections of MSC-derived exosomes did not induce toxicity in mice (Mendt et al., 2018). Repeated MSC-derived exosomes injections were well tolerated and showed no substantial side effects in patients with graft-versus-host disease (Kordelas et al., 2014).

By far, some results indicated the potential therapeutic link between exosomes and NLRP3. Injected αv integrin-specific RGD (R, arginine; G, glycine; D, aspartic acid)-modified peptide on engineered natural exosomal DOX delivery platform exhibited therapeutic response in mammary tumor mice model (Kim et al., 2016). DOX is cytotoxic drug targeting tumors that could induce pyroptosis by activating NLRP3 inflammasomes (Antonopoulos et al., 2013; Zeng et al., 2020; Wu et al., 2021), thus DOX-carried exosomes might exert latent therapeutic effects on regulating the NLRP3 signal pathway. In PD mouse model, after injection, blood-derived exosomes carried with dopamine were detected in the brain. And compared with free dopamine, exosomal dopamine exhibited higher therapeutic efficacy with reduced toxicity. Similarly, dopamine has been proven to control inflammation by inhibiting NLRP3 inflammasome in PD (Yan et al., 2015; Zhu et al., 2018; Cheng et al., 2020).

However, the potential therapeutic application of exosomes on NLRP3 remains to be verified in further studies.

## Conclusion

Nearly 40 years have passed since the exosomes were first discovered (Zhang et al., 2019d). Indeed, numerous basic and clinical studies have uncovered the mystery of exosomes under physiologic and pathologic conditions (Kalluri and LeBleu, 2020). Noteworthy, it has been widely recognized that exosomes have a crucial role in many diseases (Yang et al., 2020). The relationship between inflammation and exosomes in diseases has been further explored, highlighting the importance of NLRP3 inflammasome (Cypryk et al., 2018). The subsequent studies have shown that exosomes can modulate NLRP3 inflammasome, thus affecting the diseases process. Exosomal miR-148a can reduce the damage of the ischemic myocardium via the TXNIP-NLRP3-caspase-1 pathway (Vicencio et al., 2015). Other studies indicated that MSC-Exo-derived miR-126 could suppress hyperglycemia-induced inflammation by down-regulating HMGB1, which can bind to TLRs and activate the NLRP3 pathway (Zhang et al., 2019c). Profound insight into the molecular mechanisms underlying the exosome regulation of NLPR3 could promote their application in clinical treatment. In these studies on exosomes modulation, the molecular mechanisms in diseases progression were mainly confirmed by “gain-of-function” experiments, which may not be accurate enough to represent the exact functions of exosomes to the full extent. Meanwhile, the existing literature failed to identify the actual molecular pathway between exosomes and NLRP3. Therefore, the application of exosomes in the treatment of various diseases has great potential and deserves further exploring. We anticipate that more significant research will break the bottlenecks and lead to breakthroughs in clinical treatment.

## Glossary

ABHD5: abhydrolase domain containing 5
ASC: apoptosis-associated speck-like protein containing a CARD
ATP: adenosine triphosphate
CARD: c-terminal caspase recruitment domain
CD47: cluster of differentiation 47
CD63: cluster of differentiation 63
CD81: cluster of differentiation 81
CD9: cluster of differentiation 9
circHIPK3: circular homeodomain-interacting protein kinase 3: circular homeodomain-interacting protein kinase 3
DAMPs: damage associated molecular patterns
DOX: doxorubicin
Drp1: dynamin-related protein 1
EEF1A1: recombinant eukaryotic translation elongation factor 1 alpha 1
EEF2: recombinant eukaryotic translation elongation factor 2
ES-Exo: embryonic stem cell-derived exosomes
EVs: extracellular vesicles
FABP5: fatty acid-binding protein 5
FOXO3a: fork head box class O 3a
GELNs: ginger ELNs
GP-63: glycoprotein 63
GSDMD: gasdermin D
HMGB1: high mobility group box-1
Hsp: heat shock proteins
I/R: ischemia/reperfusion
IL-18: interleukin-18
IL-1β: interleukin-1β
ILVs: intraluminal vesicles
IPF: idiopathic pulmonary fibrosis
LLNs: liposome‐like nanoparticles
lncRNA: long non-coding RNA
LPS: lipopolysaccharides
LRR: leucine-rich repeat domain
MAVS: mitochondrial abtiviral-signaling protein
MHC: major histocompatibility complex
miRNAs: microRNAs
mRNAs: messenger RNAs
MSC: mesenchymal stem cell
mtROS: mitochondrial reactive oxygen species
MVB: multivesicular bodies
NACHT: nucleotide-binding and oligomerization domain
NASH: non-alcoholic steatohepatitis
NCMs: neonatal rat cardiomyocytes
NEK7: NIMA related kinase 7
NF-κB: nuclear factor κ B
NLR: NOD-like receptor
NLRP3: NOD-like receptor family, pyrin domain containing 3
ox-mtDNA: oxidized mitochondrial DNA
P2X7R: purinergic ligand-gated ion channel 7 receptor
PAMPs: pathogen-associated molecular patterns
PC: prostate cancer
PD: Parkinson’s disease
PD1: programmed death-1
PD-L1: programmed death-ligand 1
PPAR: peroxisome proliferator-activated receptor
PTEN: phosphate and tension homology deleted on chromsome ten
PYD: N-terminal pyrin domain
ROS: reactive oxygen species
S100-A9: S100 calcium binding protein A9
SIRPα: signal regulatory protein α
SIRT1: sirtuin 1
TAMs: tumor-associated macrophages
TLRs: toll-like receptors
TME: tumor microenvironment
TRIM59: Tripartite motif-containing 59
TXNIP: thioredoxin-interacting protein
UMSC-Exo: umbilical cord mesenchymal stem cells-derived exosomes
